# Histological Diagnosis of Madura Foot (Mycetoma): A Must for Definitive Treatment

**DOI:** 10.4103/0974-777X.52985

**Published:** 2009

**Authors:** Kiran Alam, Veena Maheshwari, Shruti Bhargava, Anshu Jain, Uroos Fatima, Ershad ul Haq

**Affiliations:** *Department of Pathology, Jawaharlal Nehru Medical College, Aligarh Muslim University, Aligarh, India*; 1 *Department of Taif, Kingdom of Sanudi Arabia*

**Keywords:** Actinomycetoma, Eumycetoma, Madura foot

## Abstract

Mycetoma, an uncommon chronic infection of skin and subcutaneous tissues in tropical countries is caused by true fungi (eumycetoma) or by filamentous bacteria (actinomycetoma). Since the treatment of these two etiologies is entirely different, a definite diagnosis after histopathological and microbiological examination is mandatory. We hereby present five cases of Madura foot.

## INTRODUCTION

Mycetoma is an uncommon chronic infective disease of the skin and subcutaneous tissues characterized by the triad of tumefaction, draining sinuses and presence of colonial grains in the exudates.[1–3] It is predominantly a disease of tropical countries and is named after the region of India where it was first described in 1842.[4] The most common site of occurrence is foot (approximately 70% cases), which explains the synonym “Madura foot”.[5] Hand is the next most common site. Repeated minor trauma or penetrating injury provides a portal of entry for the organism. Infection can be caused by true fungi in 40% cases where it is known as eumycetoma and by filamentous bacteria of order actinomycetes (actinomycetoma) in 60% cases.[4] Therapy of these two groups is entirely different,[6] thereby necessitating the need to differentiate between -the two in cases of patients presenting with Madura foot. Here we are describing five cases of Madura foot that were correctly diagnosed after detailed clinical, histopathological and microbiological studies.

## CASE REPORT

**Figure 1:**
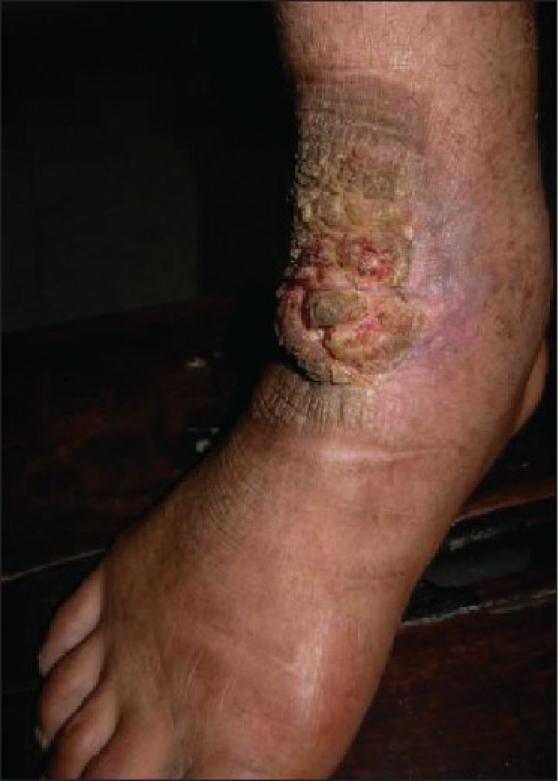
Madura foot-partially healed ulcer on the dorsal surface of ankle joint

Five male patients in the age range of 30-50 years presented in the surgical clinics with chief complaints of ulcer foot with purulent draining sinuses. Of the three patients presenting with ulcer, one showed features of healing [Figure 1]. The other two cases gave history of purulent discharge admixed with black granules. All patients belonged to rural backgrounds and had history of walking barefoot as necessitated by their occupational needs. Clinically all were diagnosed as Mycetoma foot or “Madura Foot”, and were advised biopsy along with culture of the purulent discharge. Crush smears of the discharged black granules were also prepared.

Grams' staining was performed in all cases which helped in distinguishing between actinomycetoma (Gram positive) and eumycetoma (Gram negative).

All these cases were subjected to biopsy. Grossly indurated skin with central ulceration surrounded by granulation tissue was received. On cut section subcutaneous abscess with multiloculations was seen.

**Figure 2:**
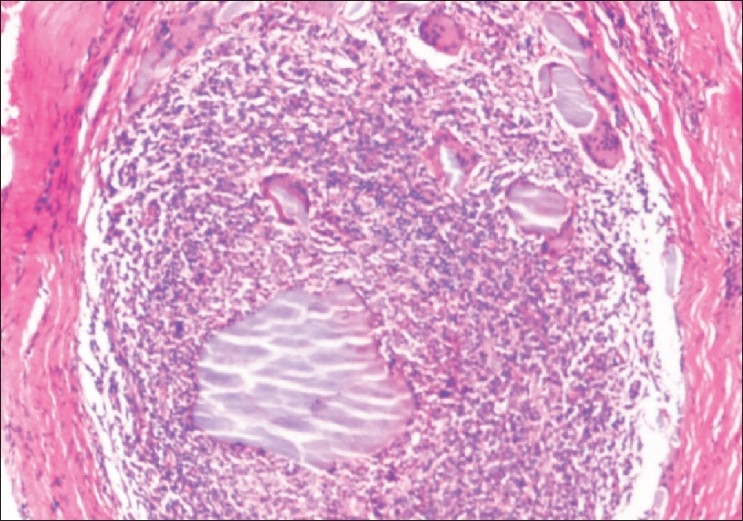
Actinomycetoma-neutrophilic infiltrate surrounding the actinomycotic colony with occasional giant cell (H and E, ×250)

**Figure 3:**
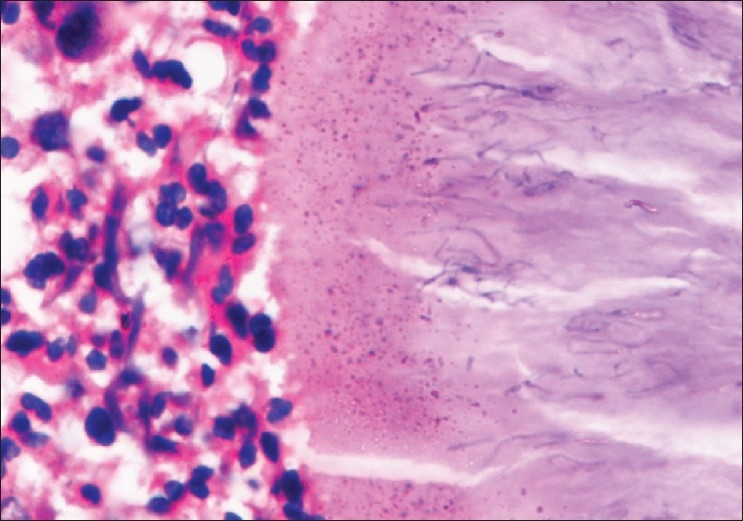
Actinomycetoma-thin filamentous bacteria seen (H and E, ×500)

**Figure 4:**
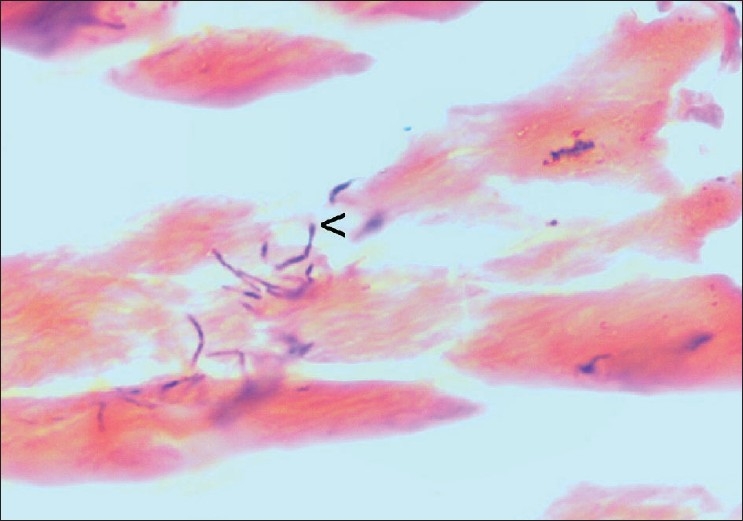
Eumycetoma-thick club shaped structures (<) (H and E, ×1250)

Histologically, H and E stain showed suppurative granulomas (composed of neutrophils) surrounding characteristic grains in the subcutaneous tissue. Neutrophilic infiltrate was seen surrounded by palisading histiocytes beyond which a mixed inflammatory infiltrate comprising lymphocytes, plasma cells, eosinophils, macrophages and fibrosis was seen. The occasional multinucleated giant cell was also seen [Figure 2]. H and E stained sections and Gram stain showed thin filamentous bacteria in cases of actinomycetoma [Figure 3] and thick club shaped structures in eumycetoma [Figure 4].

## DISCUSSION

Mycetoma is endemic in the tropics and subtropics, namely Africa, Mexico and India, and is named after the region of India where it was first described in 1842.[4] However, it can also be found in natives of areas of Central and South America and the Middle or Far East between latitudes 15°S and 30°N. The incidence of mycetoma is likely to rise in temperate regions as well, due to increases in worldwide travel. It commonly presents between 20 to 50 years of age, with a male to female ratio of 2.2:1.[4]

Mycetoma typically presents in agricultural workers or in individuals who walk barefoot in dry, dusty conditions. Minor trauma allows pathogens to enter the skin from the soil.[6] The two main etiological groups of mycetoma-actinomycetic mycetoma and eumycetic mycetoma are caused by a number of species. Over 30 species have been identified to cause mycetoma.[4,7] Actinomycetoma is caused by a group of filamentous bacteria, among which *N. brasiliensis* and *Streptomyces madurae* are the most common. In rare instances *N. asteroides*, commonly the cause of pulmonary nocardiosis and *Actinomyces israelli*, commonly the cause of Actinomyces produce a mycetoma on the feet or hands.[3,8,9] Eumycetoma is caused by a group of fungi with thick, septate hyphae, including *Allescheria boydii*, *Madurella griesia* and *Madurella mycetomi.*[9]

The grains discharged from the sinuses vary in size, color and consistency [Tables 1 and 2], features that can be used for rapid provisional identification of the etiological agent. [4] Grains of many species have overlapping morphological features and therefore culture is required for accurate identification of the causal agent. The size of grains varies from microscopic to 1-2 mm in diameter. Large grains are seen with madurellae, *Actinomadura madurae* and *A. palletieri* whereas granules of *N. brasiliensis*, *N. cavae* and *N. asteroides* are small.[10] Dark (black) grains are found only among the eumycotic mycetoma. The pigment is a melanoprotein or related substance.[11] The consistency of most grains is soft but those of *Streptomyces somaliensis* and *Madurella mycetomatis* can be quite hard.[10]

**Table 1:** The color of the grains in mycetomas and related species

Color of grains	Species
Eumycetoma - black grains	*M. mycetomatis, M. grisea, Leptospheria senegalensis, Exophiala jeanselmei, P. romeroi, C. lunata, Phialophora verrucosa, P. parasitica*
Eumycetoma - pale grains	*P. boydii, Aspergillus nidularis, A. flavus, Fusarium Sp, Acrimonium Sp, Neotestudina rosatii, dermatophytes*
Actinomyetoma - red grains	*Actinomadura pelleitieri*
Actinomycetoma - yellow grains	*Streptomyces somaliensis*
Actinomycetoma - pale grains	*N. brasiliensis, N. cavae, N. asteroides, Actinomadura madurae*

**Table 2:** Morphology of the grains in mycological and related species

Species	Morphology of grains
Eumycetomas	
Madurella mycetomatis	Large granules (up to 5 mm or more) with interlacing hyphae embedded in interstitial brownish matrix; hyphae at periphery arranged radially with numerous chlamydophores.
Petriellidium boydii	Eosinophilic, lighter in the centre; numerous vesicles or swollen hyphae; peripheral eosinophilic fringe; other pale eumycetomas have a minimal fringe and contain a dense mass of intermeshing hyphae.
Actinomycetomas	
Actinomadura madurae	Large (1-5 mm and larger) and multilobulate; peripheral basophilia and central eosinophila or pale staining, filaments grow from the peripheral zone.
Streptomyces somaliensis	Large 0.5-2 mm or more) with dense thin filaments; often stains homogenously; transverse fracture lines.
Nocardia brasiliensis	Small grains (approximately 1 mm); central purple zone; loose clumps of filaments;
	Gram positive delicate branching filaments breaking up into bacillary and coccal forms; Gram negative amorphous matrix.

The incubation period varies from several weeks to months.[4] Sinuses develop after 6-12 months and extension to involve the underlying fascia, muscle and bone is common. Rarely there is lymphatic dissemination to regional lymph nodes.[12] Actinomycetic mycetomas expand faster, are more invasive and have more sinuses than eumycotic variants.[4]

A Gram stain is of considerable value in distinguishing between actinomycetoma and eumycetoma; the fine, branching filaments, only about 1 micron thick, within the grains of actinomycetoma are gram-positive, whereas the grains of eumycetoma are gram negative.[3,10] The filaments and hyphae of the causal agent can be stained better in biopsy samples with Gram stain (actinomycetoma) or Gomori methenamine silver or periodic acid-Schiff stains (eumycetoma). It can then be seen that the granules of actinomycetoma consist of fine, branching filaments, only about 1 micron thick, whereas the granules of eumycetoma are composed of septate hyphae 4-5 microns thick.[13] The study of discharged granules crushed on slide and stained with lacto-phenol blue particularly allows differentiation between the thin filaments of actinomycetoma and the thicker hyphae of eumycetoma.[3] Hence, these special stains are of value to further confirm the nature of the organism.

It is important to let the lab know about the organism suspected because most bacterial pathogens grow out overnight but actinomycetes takes longer to be visible on the culture plates (48-72 hrs). There are no serological tests for specific diagnosis of the species.[9] In tropics this disease may go undiagnosed or untreated for so long that surgical amputation may be the only effective treatment.

The main differential diagnoses are chronic bacterial osteomyelitis, tuberculosis, or the early phase of Buruli ulcer. Other deep fungal infections such as blastomycosis or coccidiodomycosis and also leishmaniasis, yaws and syphilis should be considered. Differentiation between actinomycetoma and eumycetoma is important because of the different responses to treatment. Surgical debridement, followed by prolonged appropriate antibiotic therapy for several months is required for actinomycetoma, wherein a combination therapy with trimethoprim-sulfamethoxazole, dapsone and streptomycin has been used along with Rifampicin for resistant cases.[14]

Eumycetomas are only partially responsive to anti-fungal therapy but can be treated by surgery due to their normally well circumscribed nature. Surgery in combination with azole treatment is the recommended regime for small eumycetoma lesions in the extremities. *Madurella mycetomatis* may respond to ketoconazole; *P. boydii* (*S. apiospermum*) may respond to itraconazole. Other agents of eumycetoma may respond intermittently to itraconazole or amphotericin B.[15]

## CONCLUSION

Due to slow and relatively pain free progression of the disease, mycetoma is often at an advanced stage when first diagnosed. Prognostically, actinomycetoma can be cured with surgical debridement and appropriate antibiotic therapy while eumycetoma is only partially responsive to antifungal agents, has high rate of recurrence and may require amputation. A high incidence of secondary bacterial infection in mycetoma lesions has been reported,[16] which can cause increased pain and disability as well as septicemia which may be fatal if untreated. This emphasizes the need for its correct diagnosis after meticulous clinical examination, assisted by histological and microbiological studies along with the use of special stains.
